# Revealing Adsorption Behaviors of Amphoteric Polyacrylamide on Cellulose Fibers and Impact on Dry Strength of Fiber Networks

**DOI:** 10.3390/polym11111886

**Published:** 2019-11-15

**Authors:** Xinyu Zhang, Yangyang Zhu, Xiaoyan Wang, Peipei Wang, Jing Tian, Wenyuan Zhu, Junlong Song, Huining Xiao

**Affiliations:** 1 Jiangsu Co-Innovation Center for Efficient Processing and Utilization of Forest Resources, Nanjing Forestry University, Nanjing 210037, China; zhangxinyu4391@163.com (X.Z.); gnawxy@163.com (X.W.); peipeiwang0305@163.com (P.W.); jingtian@njfu.edu.cn (J.T.); ppzhuwy12@gmail.com (W.Z.); 2 China Light Industry Wuhan Design and Engineering Co. Ltd, Wuhan 430060, China; zhuyy1636@qgsj.com; 3 Jiangsu Provincial Key Lab of Pulp and Paper Science and Technology, Nanjing Forestry University, Nanjing 210037, China; 4 Department of Chemical Engineering, University of New Brunswick, Fredericton, NB E3B 5A3, Canada; hxiao@unb.ca

**Keywords:** amphoteric polyacrylamide, polyampholyte, quartz crystal microbalance with dissipation monitoring (QCM-D), adsorption, conformation

## Abstract

Amphoteric polyacrylamide (AmPAM) has been widely used in a variety of industrial areas and the adsorption behavior of AmPAM plays a crucial role in its applications. In this study, a series of AmPAMs with various molecular weights (MW) were synthesized; and their impact on dry strength of fiber networks or paper was assessed. The results showed that the optimal MW of AmPAM for strength enhancement ranged between 300 and 500 k. More importantly, the adsorption behaviors of three typical AmPAM samples on silica (model substrate) and cellulose surfaces were revealed using a quartz crystal microbalance with dissipation monitoring (QCM-D) in situ and in real time. The adsorption dynamics of AmPAM and the conformation of the adlayers were further derived. The results indicated that a relatively high adsorption amount was achieved under the conditions of a high polymer concentration, a medium pH close to its isoelectric point (IEP), a mild ionic strength, and a high charged surface; whereas the MW of AmPAM had little effect on the equilibrium adsorption mass of AmPAM, but significantly affected the conformation of adsorbed layer on substrates. Based on the adsorption behaviors of AmPAM, the explanation of the best dry strength achieved in a narrow range of MW of AmPAM is proposed. It was concluded that the appropriate balance between bridging and flocculation, penetration into fiber pores, and conformation were only achieved in the optimal MW range of AmPAM. The findings obtained from in this work enable us to better understand the adsorption behaviors of polyampholyte, and provide a guideline on molecular design of AmPAM and its applications from both fundamental and practical points of view.

## 1. Introduction

Amphoteric polyacrylamide (AmPAM) is a typical polyampholyte, carrying both basic groups and acid groups along the molecular backbone chain [1]. Due to the unique structure and properties, AmPAM has been used in a broad range of applications involving wetting, lubrication, adhesion, colloidal stability control, drug delivery and gene therapy, and paper strength improvement [2,3,4,5,6,7,8,9,10,11,12]. In paper manufacture, AmPAM exhibits much better performance in strength improvements than the addition of simple polyelectrolytes [3,13,14,15,16,17,18,19]. This is because in aqueous solution, the acid groups of AmPAM dissociate and adsorb cations from white water at the wet-end, while the basic groups dissociate and anchor onto anionic fibers [18]. 

There is a strong need for understanding the adsorption phenomena, especially in the case of polyampholytes, that will benefit the design of new functional materials and performance enhancement. Despite the existence of several theoretical and computational reports on polyampholyte behaviors at solid interfaces [20,21,22,23,24,25,26,27], there is a lack of experimental data to confirm the proposed theories and to directly allow the elucidations of the complex polyampholyte adsorption phenomena [28,29]. Israelachvili and co-workers [30] investigated the adsorption behavior of the polyampholyte polypeptide, gelatin, on a negatively charged mica surface as a function of pH and salt concentration. Then, we reported the relation between polyampholyte aqueous solution properties and their adsorption behaviors on silica and regenerated cellulose surfaces in our previous work [31]. The adsorption behaviors of four AmPAMs carrying different charge densities but with same nominal ratio of positive to negative segments and two structurally similar polyelectrolytes (a polyacid and a polybase) were investigated by using a quartz crystal microbalance with dissipation monitoring (QCM-D) technique. The results indicated that charge density of AmPAMs and charge density of substrates played a crucial role in adsorption, to control the adsorption dynamics, as well as the equilibrium adsorption amount. All samples used in the adsorption were with similar molecular weight (MW) of approximately 3000 k. However, super high MW does not necessarily improve paper tensile strength [32] and potentially induces the macroflocculation of fibers which is undesirable in terms of paper formation. In this work, a series of AmPAMs with various MW were synthesized and their performance was assessed in order to explore the optimal MW range for dry strength improvement of fiber networks or paper sheets.

Quartz crystal microbalance (QCM) is one of the noninvasive methods which have been used extensively to monitor the adsorption of polymers and surfactants on different surfaces. The mechanical oscillatory nature of QCM resonators could analyze the viscoelasticity adsorbed polymer films [33,34,35,36]. In the current work, a quartz crystal microbalance with dissipation monitoring (QCM-D) was employed to study the adsorption behaviors of three AmPAM samples with different MWs under various conditions (i.e., substrates, polymer concentration, media pH, and ionic strength) in an attempt to reveal the relationship between adsorption behaviors and dry strength of fiber networks from a fundamental point view. 

## 2. Materials and Methods

### 2.1. Materials

Monomeric acrylamide (AM), metharoycholine (DMC), and itaconic acid (IA) were obtained from Sinopharm Chemical Reagent Co., Ltd (Shanghai, China), Tokyo Chemical Industry Co., Ltd. (Tokyo, Japan), and Tianjin Chemical Reagent Co., Ltd. (Tianjin, China), respectively. Microcrystalline cellulose (MCC) and sodium bisulfite were purchased from Sinopharm Chemical Reagent Co., Ltd., too. Polyethyleneimine (PEI, with *M*_w_ ~ 750,000, 50 wt.% in H_2_O) was purchased from Sigma-Aldrich Co. LLC (Shanghai, China). The rest of the chemicals were ordered form Nanjing Chemical Reagent Co., Ltd. (Nanjing, China). All chemicals are analytical grade and used without further purification. QCM-D sensors were AT cut quartz crystals and were supplied by Biolin Scientific Co., Ltd. (Gothenburg, Sweden) with silica surface.

### 2.2. Synthesis of AmPAM Polymers

The procedures of AmPAM synthesis were addressed in detail in the literature [18]. Free radical copolymerization was carried out in a four-neck flask equipped with a reflux condenser. The molar ratio of monomers was 86% AM, 10% DMC, and 4% IA to ensure that all samples had the similar charge density and the same ratio of positive to negative charged groups. The initiator consisted of ammonium persulfate and sodium bisulfite (mass ratio of 5:4). First, sodium bisulfite was added, then the ammonium persulfate was added dropwise 10 min later, followed by polymerization at 60 °C for 4 h. Solid AmPAM samples were obtained after precipitation with acetone, followed by drying at 60 °C in a vacuum oven. By changing the reaction parameters, like temperature, medium pH, initiator charges, and concentration of monomers, a series of AmPAM polymers with different MW were obtained.

The viscosity MW of AmPAM was checked according to Chinese standard GB/T12005.10-9. A 0.1% solution of AmPAM in 1 M NaCl solution was prepared initially. The viscosity of aqueous AmPAM solutions was measured using an Ubbelohde viscometer (Nanjing, China) in a water bath at 30 °C. The flow times of AmPAMs and the 1 M NaCl solutions in the viscometer were recorded. The viscosity-average molecular weight of AmPAM was calculated according to Equation (1), where [η] represents intrinsic viscosity.

MW = 802[η]^1.25^ (1)

The MW of AmPAM polymers obtained were checked and their MW ranged from 5.4 to 713 k.

### 2.3. Preparation of Handsheet Treated with AmPAMs and Strength Characterization

Bleached hardwood kraft fibers were provided by Asia Symbol (Shandong, China) and beaten with a laboratory valley beater (TD6-23, Xianyang Tongda Light-Industry Equipment Co., Ltd., Xiangyang, China) to a beating degree of 35° SR (standard GB/T 3332-1982). Before handsheet preparation, 1000 mL of slurry (with 0.2% oven-dried fiber) was blended with 10 mL of 0.1 wt.% AmPAM solution (0.5 wt.% load based on fiber) and stirred for 5 min. Handsheets were prepared in an automatic rapid Köthen sheet machine mold (Frank-PTI, Birkenau, Germany) with a base weight of 68 g/m^2^. The wet handsheets were dried for 15 min. 

The breaking length (standard of GB/T 12914) of the handsheets was measured on a tensile machine (Qingtong Instrumental Co., Ltd., Hangzhou, China). Prior to measurements, handsheets were stored at 23 °C and 50% humidity for more than 48 h. The handsheets were cut into pieces of 15 mm (width) by more than 150 mm (length). The pulling force of paper sheet for tensile failure was recorded to calculate the breaking length. 

### 2.4. Preparation of Cellulose Model Films

QCM sensors with silica surface were cleaned by sonication in a 2% sodium dodecyl sulfate solution for 15 min and then subjected to UV-Ozone radiation (28 mW/cm^2^ at 254 nm) for 10 min, immediately before spin-coating. 

In term of regenerated cellulose (RC) film, it was prepared according to a procedure reported elsewhere [37] and modified slightly as follows. First, cellulose solution was prepared by dissolving microcrystalline cellulose (MCC) in N-methylmorpholine-N-Oxide (NMMO) aqueous solution (50 wt.%) at 115 °C. Dimethyl sulfoxide (DMSO) was added to adjust the viscosity of the cellulose solution and the concentration to 0.5%. PEI was used as anchoring polymer of the cellulose film on the surface sensors. The substrates were immersed in PEI (100 mg/L) for 20 min, followed by washing with milli-Q water and drying with the gentle nitrogen jet. The cellulose solution was then spin-coated (Model KW-4A, Shanghai Daojing Instrument Co., Ltd., Shanghai, China) by depositing 50–100 μL on the PEI-modified substrates at 3000 rpm for 60 s. The RC-coated sensors were removed from the coater and then immersed in milli-Q water for 4 h and then placed in an oven for 2 h at 80 °C. The RC-coated substrates were then stored at room temperature in a clean chamber prior to further use.

In terms of the cellulose nanocrystal (CNC) film, pristine CNC was produced at first by the procedures adopted from Beck-Candanedo’s method [38]. MCC (5 g) and H_2_SO_4_ (250 mL, 64 wt.%) were charged into a three-neck flask equipped with a mechanical stirrer. This mixture was hydrolyzed at 45 °C for 30 min under constant stirring. After hydrolysis, the suspension was subjected to centrifuge to remove excess acid for several times until the suspension appeared turbid. Then, it was transferred into a dialysis bag (Sangon Biotech, Shanghai) with molecular weight cut-off (MWCO) 14,000 to dialyze against pure water for several days until the medium pH went down to a constant close to pure water. The second step was spin-coating. The cleaning procedures and modification with PEI were the same as addressed previously. Prior to spin-coating, CNC suspension was treated by ultra-sonication for 10 min. Then, the CNC suspension was spin-coated (Model KW-4A, Shanghai Daojing Instrument Co., Ltd., Shanghai, China) by depositing one to two drops on the PEI-modified substrates at 3000 rpm for 60 min. The obtained sensors were cured in an oven for 2 h at 80 °C to stabilize the films and preventing the films from dispersing in the imminent liquid phase.

### 2.5. Adsorption Behaviors Monitored by QCM-D Technique

Adsorption of AmPAM on various substrates was conducted on a Quartz Crystal Microbalance with Dissipation mode, E4 model (Biolin Scientific Co. Ltd., Gothenburg, Sweden). The principles of this technique can be found in [39]. 

In the case of rigid film, there is a simple relationship (Equation (2)) between adsorption mass (∆*m*) and the recorded frequency change (∆*f*), which is called the Sauerbrey equation [40].

(2) $$\Delta m=-\frac{c\Delta f}{n}$$

Resonators consisting of silica-coated quartz AT-cut crystals with fundamental frequency of 5 MHz were used as bare sensors or modified with regenerated cellulose film or with cellulose nanocrystals. These crystals provided a mass sensitivity of 17.7 ng cm^−2^ Hz^−1^ for rigid layers. This system allowed for the measurement of up to seven harmonics. In this study, the frequency and dissipation responses were recorded at around 15, 25, and 35 MHz, corresponding to the third, fifth, and seventh overtones (*n* = 3, 5, and 7, respectively). The third overtone frequency and dissipation acquired by QCM-D were reported for each film.

The Sauerbrey relation was initially developed for adsorption from the gas phase but it is now extended to liquid media where it holds in most cases. In order to describe soft adlayers of polymer adsorbing from liquid media, the dissipation value *D* was introduced. Rodahl et al. [41] extended the use of the QCM technique and introduced the measurement of the dissipation factor simultaneously with the resonance frequency by switching on and off the voltage applied onto the quartz. The measured change in dissipation was originated by changes in the coupling between the oscillating sensor and its surroundings, and it was influenced by the layer’s viscoelasticity and slip of the adsorbed layer on the surface. The dissipation factor *D*, the inverse of the so-called *Q* factor, is defined by Equation (3):

(3) $$D=\frac{1}{Q}=\frac{E_{disspated}}{2\pi E_{stored}}$$

where *E_dissipated_* is the energy dissipated during one period of oscillation and *E_stored_* is the energy stored in the oscillating system.

In a typical experiment, fresh polymer solutions with the concentration of 0.1 mg/L (i.e., 100 ppm) were initially prepared with milli-Q water. Before running the polymer solutions, the instrument was stabilized with the respective solution which was used for preparing polymer solutions, called buffer solution here. After running the QCM for 10 min with buffer solution, a constant QCM baseline was obtained and thereafter 1 mL of polymer solution was injected in the adsorption module at a low rate (0.1 mL/min). After both frequency and dissipation reached stability, 3 mL of buffer solution was used to rinse the adsorbed layer (using the same injection rate). By using this rinsing procedure, any loosely bound polymer was removed from the interface and the net adsorption was, therefore, accounted for. The frequency and dissipation changes were monitored during the whole process. The temperature of the adsorption was controlled at 25 °C.

## 3. Results and Discussion

### 3.1. Dry Strength of Handsheets Treated with AmPAM of Various MWs

**Figure 1:**
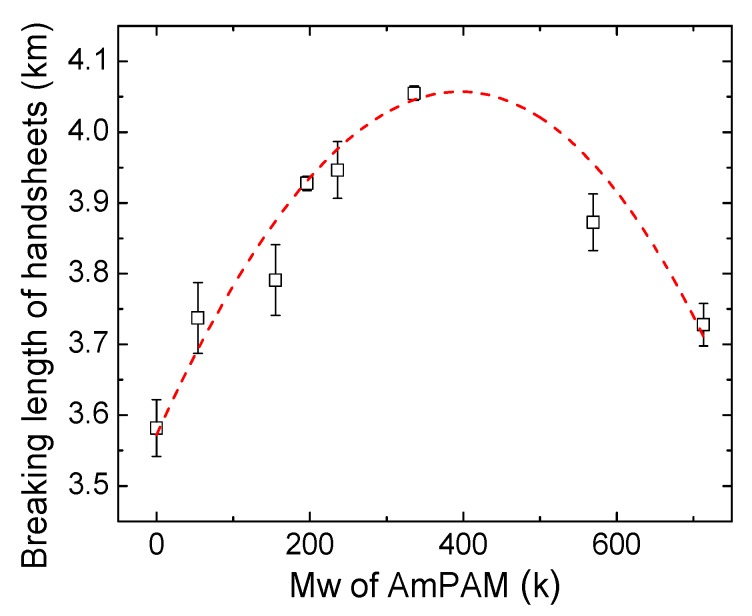
Breaking length of paper treated with amphoteric polyacrylamide (AmPAM) of various molecular weights (MWs) at 0.5 wt.% (on o.d. fiber). Polynomial regression with order 3 was presented as a red, dashed line.

Handsheets were prepared by adding 0.5% AmPAM (wt. on over-dried or o.d. fiber) to fiber slurry. The dry strength was measured and expressed as the breaking length of paper or fiber networks. The results were plotted against AmPAM MW; and are shown in Figure 1. The breaking length of the control sample was 3.57 km. In the presence of AmPAM with MW of 54 k, the breaking length increased by 4% to about 3.73 km. With the increase of the MW up to 336 k, the dry strength kept increasing. In other words, the optimal dry strength of paper was achieved with the addition of AmPAM with MW of 336 k, allowing the breaking length to reach 4.05 km, which was 15.0% higher than that of the control. However, further increasing the MW of AmPAM above 336 k, the trend was overturned and the breaking length decreased with increasing MW. The dashed line in Figure 1 was the polynomial regression of the trend between breaking length of the handsheets and MW of AmPAM with an order of 3. From this curve, it is clearly demonstrated that there existed a plateau for the MW of AmPAM in the range of 300 to 500 k, which led to the optimal dry strength of paper. The adsorption behavior of AmPAM plays a crucial role in its performance. In order to elucidate how the MW of AmPAM influences the dry strength of the handsheet, the adsorption behaviors of three typical AmPAM samples with MW of 196, 336, and 713 k were selected to represent low, optimal, and high MW, respectively. The adsorption tests were conducted on the surfaces of silica and cellulose, and dynamically monitored using a quartz crystal microbalance with dissipation monitoring (QCM-D) in situ and in real time.

### 3.2. Adsorption Behaviors of AmPAMs

#### 3.2.1. Effects of Molecular Weight on the Adsorption Behaviors of AmPAMs

Among AmPAM samples synthesized, three samples with MW of 196, 330, and 713 k were picked out as representatives to conduct the QCM-D experiments to investigate their adsorption behaviors on model surfaces (i.e., silica, regenerated cellulose, and cellulose nanocrystal surfaces).

**Figure 2:**
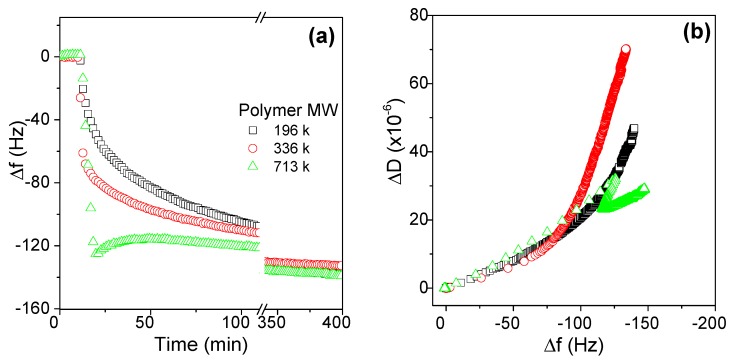
Effects of molecular weight of AmPAM samples on the adsorption amount (**a**) and conformation (**b**) of the AmPAM adlayers on silica substrate. Three tested AmPAM samples were of MW of 196, 336, and 713 k, respectively. The AmPAM solutions were of concentration of 100 mg/L and ionic strength was controlled at 0.02 M.

The adsorption behaviors of three AmPAM samples with the same charge ratio but with different molecular weights on silica surface are shown in Figure 2. It shows that the adsorption masses at plateau were roughly the same, corresponding to about −140 Hz, even though there were apparent differences in adsorption behavior in the initial stage of the adsorption. At the beginning of the adsorption, the higher the molecular weight, the faster the adsorption speed. However, the conformation of the adlayers in the initial stage, i.e., the frequency change was greater than −100 Hz, was almost the same regardless of MW, as shown in Figure 2b. The difference in conformation occurred when the frequency change was lower than −100 Hz. For the samples with MW of 196 and 336 k, the conformation of the adlayer became softer, while for the sample with MW of 713 k, its conformation of the adlayer became more compact since there was an arrangement occurring at the frequency change of −120 Hz. Why the high MW AmPAM led to this phenomenon still needs to be further investigated. 

To reveal the influence of polymer molecular weight or chain length on its adsorption amount on cellulose fibers, Wågberg et al. [42] compared the adsorption masses of three cationic polyelectrolytes (polydimethyldiallylammonium chloride, PDMDAAC) with MW 8.75 k (LMw), 48 k (MMw), and 1200 k (HMw), respectively, onto bleached chemical fibers; and the amount of adsorption was measured by polyelectrolyte titration. They found that the adsorption amount of MMw was equal to that of HMw, and adsorption took place on the external surface of fibers. LMw adsorption was much higher due to the adsorption of polymer chains on the internal surface of fibers as well. Therefore, molecular weight does not affect the adsorption mass of AmPAM if there is no polymer penetration into the inner porous structure of cellulose fibers. In our case, the substrate used in the investigation was silica; therefore, the polymer penetration would not happen due to non-porous silica selected. Therefore, the similar adsorption mass for three AmPAMs with different MW would be expected if there was only electrostatic forces involved; presumably the configuration of polymer chains on the surface of silica remains the same. 

AmPAM molecules tend to be a string shape in aqueous solution, and the string length increases as asymmetry decreases or net change increases [1]. AmPAM chains look like necklaces, and the ratio of the amount of beads to necklaces depends on the ratio of net charge and chain length. In our case, all three tested AmPAM samples had the same charge density, indicating that they had the same ratio of net charge and string length. Therefore, they should have had the same shape, and the same adsorption amount and conformation of adlayer could have been expected under the same polymer concentration. However, we all can observe that MW or chain length had some impact on the adsorption dynamics in the initial stage and on the conformation when the adlayer was thicker than a certain range. The explanation may be due to the asymmetric distribution of charged groups in the skeletons. In the above discussion, all samples were randomly synthesized and it was supposed all charged groups were randomly distributed in the chains. However, the reaction activity of cationic, anionic, and the neutral monomers should not be the same. Therefore, the distribution of charged groups may also have impacts on the conformation of adlayer and the conformational rearrangement of the chains to some extent.

#### 3.2.2. Effects of Polymer Concentration on the Adsorption Behaviors of AmPAMs

**Figure 3:**
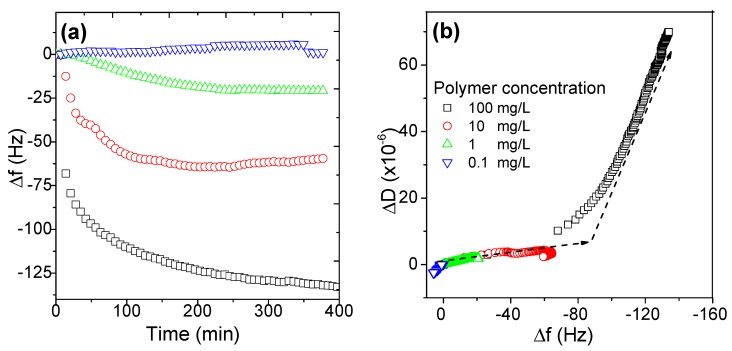
Effects of polymer concentration on the adsorption amount (**a**) and on the conformation (**b**) of the AmPAM adlayers on silica surface. The tested AmPAM sample with MW of 336 k, concentration ranging from 0.1 to 100 mg/L, and the ionic strength was controlled at 0.02 M.

Polymer concentration is the main driving force of adsorption dynamics. Effects of polymer concentration ranging from 0.1 to 100 mg/L on the adsorption amount, expressed in frequency change, are shown in Figure 3a. When polymer concentration was 0.1 mg/L, the variation of the frequency was within several Hz, indicating the adsorbed amount was very limited and the frequency change may be attributed to the noise and systemic frequency shift. When polymer concentration was above 1 mg/L, the frequency dropped obviously. The frequency drop for polymer concentration of 1, 10, and 100 mg/L was −20, −65, and −130 Hz, respectively. As can been seen from Figure 3, it was obvious that the concentration not only influenced the maximal adsorption amount strongly, i.e., the plateau of adsorption, but also the time to reach plateau, i.e., dynamics. 

The adsorption curves in Figure 3a were replotted as the dissipation value change against frequency change, Δ*D* vs. Δ*f* in Figure 3b. A very interesting phenomenon could be noted that all the curves were overlaid and two distinct slopes existed in the adsorption of AmPAM. The slope of Δ*D* and Δ*f* was an indicator of the conformation of adlayer. From this point of view, when the adsorption mass was lower than the amount expressed in frequency of −80 Hz, a relatively compact AmPAM layer was deposited on the surface no matter what concentrations were used. When the adsorption amount was greater than the critical value expressed in frequency drop, −80 Hz, the outer layer of AmPAM was formed but with a much softer conformation. The adsorption of polyampholytes tends to form a hard core and then a soft corona; this observation was similar with that of very high MW of AmPAM in our previous finding [31].

#### 3.2.3. Effects of Medium pH on the Adsorption Behaviors of AmPAMs

The amount of dissociated basic groups and acid groups along AmPAM chains in aqueous solution depends on the pH of media. The basic groups tend to be dissociated at acid condition, while acid groups at basic condition. The net charge changes with the pH of media of three samples tested in this investigation were reported in [18]. The isoelectric points (IEPs) of three samples were determined; and the similar IEPs, close to pH 7, were observed. 

**Figure 4:**
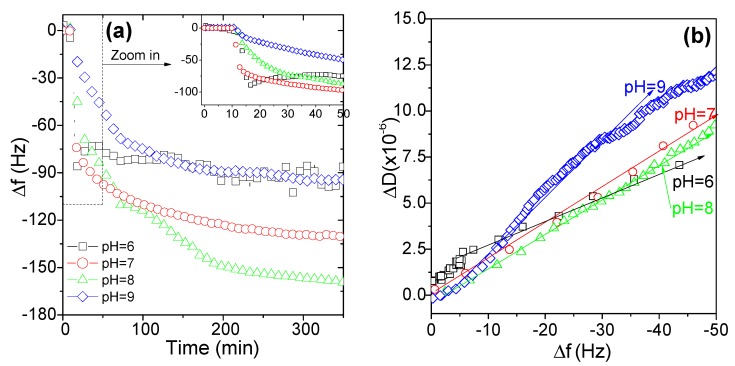
The adsorption behaviors of AmPAM on silica surface at different pH media (**a**) and the according conformation information revealed by the relationship of Δ*D*/Δ*f* (**b**). The tested AmPAM sample was of Mw of 336 k and the polymer concentration was 100 mg/L. Ionic strength of solution was controlled at 0.02 M.

The adsorption of a selected sample of AmPAM with Mw of 336 k on silica surface was conducted at different pHs, ranging from 6 to 9. The frequency change corresponding to the adsorbed amount at different pH was plotted against acquiring time; and the results are shown in Figure 4a. Meanwhile, the according conformation information of the adsorbed layers revealed by the relationship of Δ*D*/Δ*f* relationship is presented in Figure 4b. 

As can be seen from Figure 4a, the maximum adsorption was reached at pH 8, followed by the one at pH 7. While at pH 6 and 9, the adsorbed mass was similar and the least among all the four experiments conducted. Since the IEP of AmPAM samples was around pH 7, the maximal adsorption was always achieved at the condition of neutral state. The amount of adsorption is closely related to the number of layers adsorbed. The more the number of adsorbed layers, the greater the quantity of adsorption. As the adsorption progressed, the number of adsorption layers increased, and the junction force between the adsorption layers began to decrease. When it was small to a certain extent, the layer adsorption was no longer continued. At the IEP of AmPAM, the positive and negative charges were of equal amount along polymer chains, i.e., the charges were stoichiometrically equivalent. There were many bonding points between adjacent adsorption layers, and the binding force was strong enough to continue adsorbing the molecules. This trend was consistent with the observation in literature [43]. At pH 9, even though both the net charge of AmPAM and the silica surface demonstrated negative charge, it still showed comparative adsorption as that at pH 6. This was due to the unique properties of polyampholyte which can rearrange its conformation upon adsorbing on a surface even with the liked net charges. This was also consistent with our previous observations [31].

The insert in Figure 4a shows the adsorption dynamics in initial 50 min. The slopes of curves represented adsorption speed. At pH 6 and 7, the steepest slope was observed, and the smoothest one was observed for pH 9; while that of pH 8 just laid between them. At the beginning stage of adsorption, electrostatic forces dominated. For the case of pH 6, the net charge of AmPAM was positive while the silica surface was negatively charged; therefore, they had a fast adsorption speed due to electrostatic attraction. In term of pH 7, the net charge of AmPAM was close to zero and AmPAM molecules tended to form compact coils and deposit on the surface. At pH 9, as explained in the previous section, both polymer and surface carried negative charges. Even though AmPAM polymers can change their conformation to adsorb on the surface, the transformation of conformation needed some time and hindered the adsorption process. Then it makes sense why its adsorption speed was the slowest among four conditions conducted. While for the case of pH 8, although its initial adsorption speed was not fast and the equilibrium time was the longest, its total adsorbed mass was the most at equilibrium state. Briefly, electrostatic interactions between the polymer and surface dominated in the initial stage of the adsorption process of amphoteric polymers, the maximal adsorption usually achieved where the medium pH is close to its IEP. This phenomenon was consistent with the work done by Israelachvili and co-workers [30], who investigated the adsorption behavior of the polyampholyte on a negatively charged mica surface as a function of pH.

The conformation of adlayers of AmPAM characterized by curves of Δ*D*/Δ*f* is shown in Figure 4b. It is interesting to note that the conformation of adlayers of AmPAM was different on silica surface at different pH conditions. The slope of Δ*D*/Δ*f* for pH 6 was the lowest, while for pH 9 it was the highest, and for pH 7 and 8 it was in between. Based on the discussion on the electrostatic interactions between polymers and surface, the stronger interaction led to a compact adlayer while the repulsive force at pH 9 led to a softer adlayer. From this point of view, electrostatic interaction plays an important role in the determination of the structure of the adlayers of polyampholytes.

#### 3.2.4. Effects of Ionic Strength on the Adsorption Behaviors of AmPAM

**Figure 5:**
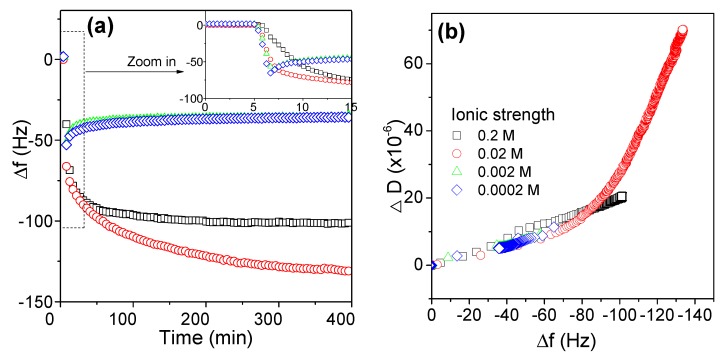
Effects of ionic strength on the adsorption amount (**a**) and the conformation (**b**) of the AmPAM adlayer. The tested AmPAM sample was of MW of 336 k and the polymer concentration was 100 mg/L.

In addition to pH changes, polyampholytes are also highly responsive to the addition of salt. The effects of ionic strength on the adsorption behaviors of AmPAM on silica surface are shown in Figure 5; and the effects on adsorption amount and conformation are presented in (a) and (b), respectively. NaCl was added to adjust the ionic strength of media. When it was very low, i.e., 0.0002 M and 0.002 M, the adsorption curves of AmPAM were almost overlapped. Increasing it to 0.02 M and 0.2 M, another interesting phenomenon was observed, i.e., the most adsorbed mass did not occur at 0.2 M (the strongest ionic strength) tested in this investigation but at 0.02 M. It meant that with the further increasing of ionic strength, the adsorption amount was decreased when the ionic strength was beyond 0.02 M.

In short, ionic strength made a difference in the molecular structure of polyelectrolytes in solution. Due to electrostatic attraction between positive and negative charged groups, AmPAM molecular chains tend to crimp and aggregate at IEP, especially under weak ionic strength conditions. Because of ion screen effect, it can reduce the electrostatic forces between positively and negatively charged groups, either attraction or repulsion. While under high ionic strength conditions, molecular chains tend to expand and charged groups expose, thereby aiding solvation. This is known as the antipolyelectrolyte “salting-in” effect [6]. 

It was worth noting that in the initial stage of the adsorption dynamics of AmPAM on silica surface, e.g., by the acquiring time of 15 min, as shown in the insert of Figure 5a, the speed of adsorption in the beginning decreased with increasing ionic strength. 

In Figure 5b, there was a turning or transition point around −40~−80 Hz in frequency change in every Δ*D*-Δ*f* curve. And some curves continued to increase as such in the ionic strength of 0.02 M and 0.2 M, while some started to go back as such in curves of 0.2 mM and 0.002 M. Those transitions indicated that the adlayers of AmPAM started to rearrange when the adsorption amount reached to −40 to −80 Hz in frequency change. The explanation is as follows:

Firstly, ionic screen effect between AmPAM and charged surface weakened attraction between AmPAM and surface. Therefore, adsorption rate in high ionic strength solution was slower.

Secondly, ionic screen effect in intra-molecules contributed to the expansion of AmPAM molecular chains. When the coils of AmPAM molecules expand enough at high ionic strength, an AmPAM molecule can connect to another. Therefore, multilayer can be formed on charged surface, and adsorbed mass can continuously increase at high ionic strength. But the situation is different for low ionic strength. As we mentioned before, the attraction force between opposite charged groups in AmPAM chains tended to form a compact coil. Since QCM-D measured the wet weight of polymer layer, the sensed mass of compact coils of AmPAM decreased due to less coupled water in the layer. In our case, both the decrease in frequency change and dissipation value, therefore, were not due to polymer desorption, but due to the rearrangement of polymer chains. This could be verified by a SPR experiment to see if the desorption takes place in the low ionic strength. 

#### 3.2.5. Effects of Substrate Surface on the Adsorption Behaviors of AmPAMs

**Figure 6:**
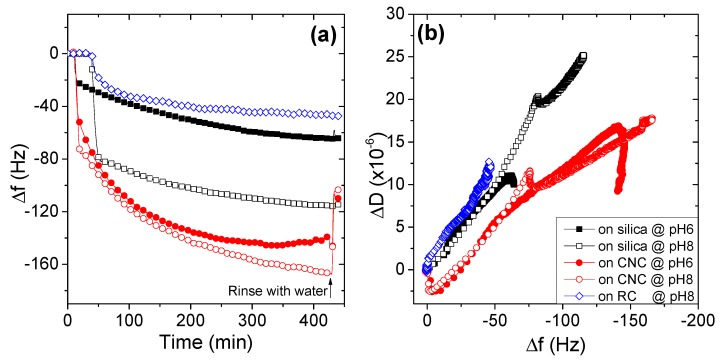
Effects of substrates and medium pH on the adsorption amount (**a**) and the conformation (**b**) of AmPAM adlayers. The tested AmPAM sample was of MW of 713 k and the polymer concentration was 100 mg/L. Ionic strength of solution was controlled at 0.02 M.

Since the adsorption took place at the interface between AmPAM solution and the substrates, the properties of both AmPAM solutions and substrate surfaces influenced the adsorption behaviors of AmPAM. In terms of substrate, two typical cellulose films were selected as model cellulose surfaces: One was regenerated cellulose (RC) film, the other was cellulose nanocrystals (CNC) film. Sometimes silica surface was used to mimic cellulose surface because it bears some negative charges on the surface [31]. The effects of substrates (silica, CNC, and RC), combined with the effects of medium pHs (6 and 8) on the adsorption amount of the AmPAM, which expressed in frequency change, and on the conformation of the adlayers are shown in Figure 6a,b, respectively. 

From Figure 6a, it can be observed that the adsorption amount of AmPAM on different substrates varied significantly. At the same medium pH 8, the frequency change on the substrate RC was −45 Hz, only about one-fourth of the amount on CNC; the frequency change on silica was about −120 Hz. At pH 6, the frequency change on silica was less than a half for CNC, i.e., −60 Hz for silica and -140 Hz for CNC, respectively. 

Another special phenomenon we observed was, for a given substrate, the adsorption amount in medium pH 8 was usually greater than that in the condition of pH 6. It implied that the greater amount of adsorption was achieved at pH close to or slightly higher than its IEP. This issue was addressed in detail for the AmPAM sample with MW of 336 k. 

In term of the conformation of adlayer of AmPAM on different substrates, as illustrated in Figure 6b, it can be found that the adlayer on CNC was the most compact, the adlayer on RC was the softest, while the adlayer on silica surface was in between. 

Khan and co-workers [44,45] investigated the effect of charge sequence of weakly coupled polyampholytes on their adsorption onto negatively charged surfaces using Monte Carlo simulations. A range of surface charge densities and degrees of polyampholyte ionization were investigated. The adsorption of polyampholytes is induced by polarization of the chain by the charged surface (known as the polarization-induced attraction mechanism). Radtchenko et al. [46] determined the charge density of colloidal silica and spin-coated cellulose film at pH 4 and 9.5, which was in the range of −0.4~−2 mC/m^2^ and −0.21~−0.80 mC/m^2^, respectively. CNC film is of the highest negative charge density due to the sulfuric ester bonds formed in the process of CNC production [47]. Therefore, the charge density of the three substrates we tested followed the sequence of CNC > silica > RC. Apparently, the adsorption amount of AmPAM on substrates follows the same order of charge density of substrates. Moreover, the conformation of adlayers followed the same trend: The higher charge density of substrate, the more compact the adlayer will be. The phenomenon that adsorption mass of polyampholytes on silica surface was much greater than that on RC surface was also observed for higher MW of polyampholytes in our previous study [31]. The results demonstrated that our observation on the adsorption behaviors of polyampholytes on charged surfaces agrees well with the computer simulation. It also implies that the charge property of the substrate plays a crucial role in the adsorption of polyampholytes. 

Numerical simulations by other groups have shed light to understand the adsorption behavior of polyampholytes on charged surfaces [44,45]. Our experimental results highlight the importance of the balance of charges, and the environmental factors which may affect the balance of charges between polyampholytes and substrate surface, (i.e., pH and ionic strength of aqueous medium). Finally, since polyampholytes are a kind analogy for protein, this investigation is also helpful for a better understanding of protein adsorption.

### 3.3. Elucidation of Impact of AmPAM MW on Dry Strength of Handsheets

Based on the observations above, it was found that a greater adsorption amount was achieved under the conditions of a high polymer concentration, a medium pH close to its IEP, a mild ionic strength and a high charged surface. All variables influencing the adsorption of AmPAM (MW and concentration of polymer, pH and ionic strength of medium, and substrate) will eventually impact on the dry strength performance of AmPAM. In our case, the optimal pH condition obtained from QCM experiments was just located in the typical range of papermaking, while other parameters were not likely to be adjusted in practice, for example, the surface charge of substrate and ionic strength of medium. Therefore, polymer and polymer structure are key parameters which are easy to adjust for the paper strength enhancement in practice. However, the MW of AmPAM had little effect on the equilibrium adsorption mass of AmPAM onto a surface, but indeed had some influence on the adsorption dynamics and the conformations of the adsorbed layer. 

The AmPAM with moderate MW led to the softest adsorbed layer or the optimal dry strength of paper, compared to those with either low or high MW. With such a MW, adsorbed layer of AmPAM appears to be more extendable and flexible, permitting more fibers to contact with polymer chains at a given dosage. From this point of view, the conformation of AmPAM with moderate MW is favorite to the dry strength.

In the meantime, the chain length of AmPAM is crucial for bridging fibers. For short polyampholytes (MW < 300 k), the breaking length of handsheets increased with increasing MW; this result was attributed to the better bridging effect of amphoteric polymers with longer chains and higher MW. When the MW of AmPAM is greater than 500 k, the polymer tends to act as a flocculant instead of a strength agent due to the excessive connection or bridging among fibers, resulting in the macroflocculation of fibers. As a result, the homogeneity of fiber networks or handsheets is reduced; and the dry strength of paper is lowered accordingly. For the AmPAM with moderate or optimal MW range, the dry strength of paper and the bridging or flocculation effects created by AmPAM are properly balanced.

Moreover, the porous structure of fiber should also be considered in the dry strength variation for AmPAM samples with different MW. For polymer with short chain [42], it has more opportunities to penetrate in the inner pores of fiber and as a consequence it should contribute little to the dry strength without connection to other fibers or itself for bonding enhancement. With a relatively high MW or a large coil size, AmPAM is difficult to penetrate in the pores of fiber and, therefore, favors the connection to fibers and polymer itself. 

To sum up the discussions above, the effect of AmPAM MW on the dry strength of paper or fiber networks is rather complicated. The AmPAM with MW in the range of 300–500 k has the best performance on enhancing dry strength due to the appropriate balance among bridging effect or flocculation, penetration into fiber pores, and conformation of polymer chains.

## 4. Conclusions

A series of AmPAMs with fixed charge ratio but various MWs were synthesized and their performance in enhancing the dry strength of handsheets or fiber networks were systematically assessed. Moreover, the adsorption behaviors of selected AmPAM samples on model silica and cellulose films were revealed using QCM-D technique to elucidate the performance of AmPAMs from a fundamental point of view. The results showed there was an optimal MW range (i.e., 300 to 500 k) for maximizing the dry strength of fiber networks. The findings obtained from QCM-D unveiled that different variables, such as polymer characteristics, charge properties of substrates, and the ionic strength and pH of medium, affected the adsorption behaviors significantly. In the current systems, the MW of AmPAM had little impact on the adsorption amount at equilibrium, but indeed influenced the conformation of adlayer of AmPAM with different MWs. Within the range of 300–500 k, the polymer chains appeared to be more expanded and favored to form interfiber connections. The findings also enabled us to better understand the correlation between the extension of adsorption and conformation of adsorbed species, which is a result of a subtle balance of influencing factors, including charge properties of surfaces, charge density of polyampholytes, and polymer charge asymmetry as well as medium properties.
